# Evaporation-induced hydrodynamics promote conjugation-mediated plasmid transfer in microbial populations

**DOI:** 10.1038/s43705-021-00057-5

**Published:** 2021-10-11

**Authors:** Chujin Ruan, Josep Ramoneda, Guowei Chen, David R. Johnson, Gang Wang

**Affiliations:** 1 College of Land Science and Technology, China Agricultural University, 100193 Beijing, China; 2 Department of Environmental Microbiology, Swiss Federal Institute of Aquatic Science and Technology (Eawag), 8600 Dübendorf, Switzerland; 3 School of Civil and Hydraulic Engineering, Hefei University of Technology, Hefei, China

**Keywords:** Microbial ecology, Environmental microbiology

## Abstract

Conjugative plasmids bestow important traits to microbial communities, such as virulence, antibiotic resistance, pollutant biotransformation, and biotechnology-relevant functions. While the biological mechanisms and determinants of plasmid conjugation are well established, the underlying physical and ecological driving forces remain unclear. Microbial communities often inhabit unsaturated environments, such as soils and host surfaces (e.g., skin, teeth, leaves, roots), where water evaporation and associated small-scale hydrodynamic processes frequently occur at numerous air-water and solid-water interfaces. Here, we hypothesized that evaporation can induce water flows with profound effects on the spatial distribution and surface deposition of cells, and consequently on the extent of plasmid conjugation. Using droplet experiments with an antibiotic resistance-encoding plasmid, we show that evaporation-induced water flows reduce cell-cell distances and significantly increase the extent of plasmid conjugation. Counterintuitively, we found that evaporation results in lower expression levels of conjugation-related genes. This negative relationship between the extent of plasmid conjugation and the expression of conjugation-related genes could be attributed to increased conjugation efficiency during evaporation. This study provides new insights into the physical and ecological determinants of plasmid conjugation, with important implications for understanding the spread and proliferation of plasmid-encoded traits.

## MAIN

Conjugative plasmids confer important traits to microbial communities, with both deleterious and beneficial effects on human health, the environment, and biotechnology [1–3]. The spread of virulence and resistance to antimicrobial agents [1, 2] and the facilitation of specific pollutant biotransformations exemplify the importance of conjugative plasmids [3, 4]. Understanding the mechanisms governing the transfer and spread of conjugative plasmids is therefore critically important. Although substantial research efforts have been made toward understanding the molecular mechanisms and biological determinants of plasmid conjugation [5], the underlying driving forces from physical and ecological aspects remain unclear.

Many microbial communities exist in environments that are periodically or continuously exposed to unsaturated water conditions. For example, the communities residing in the vadose zone of soils are periodically exposed to saturated conditions after rainfall events and irrigation, and thereafter to unsaturated conditions upon soil draining. The microbial communities inhabiting the outer surfaces of various hosts such as skin, teeth, leaves, or roots also experience frequent hydration dynamics. The air-water interfaces of such soil particles or host surfaces are subject to water evaporation when the ambient relative humidity (RH) is <100%. How does small-scale water evaporation modulate plasmid conjugation within these microbial communities?

At air-water interfaces, evaporation usually induces localized water flows that can be a driving force for passive cell movement, and thereby redistributes cells across space and affects their surface deposition [6]. Notably, the distances via evaporation-induced passive cell movement are often longer than those attributed to active cell movement (e.g., via flagella or pili) [7]. Hence, evaporation-induced passive cell movement may remarkably contribute to the spatial redistribution and surface deposition of cells in unsaturated environments. Accordingly, we hypothesize that evaporation is an important determinant of the number and duration of cell-cell contacts, and thus an important determinant of plasmid conjugation [5].

**Fig. 1:**
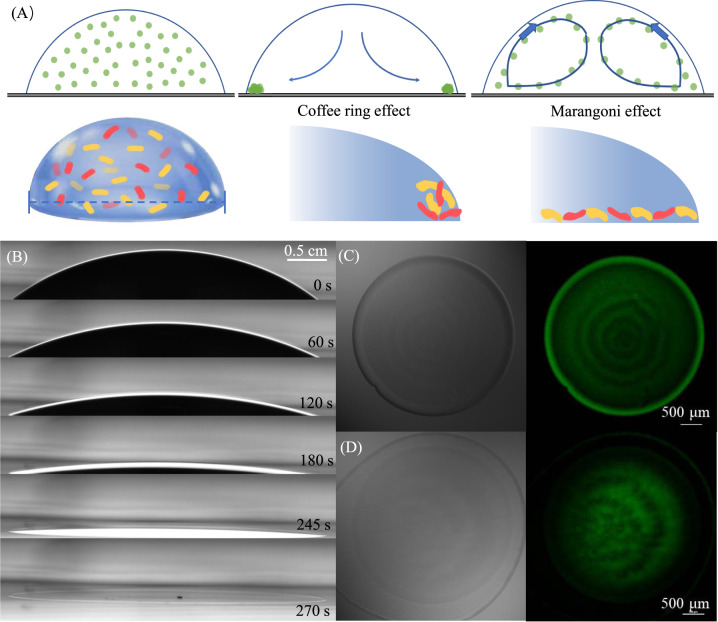
Effects of evaporation-induced water flow on passive cell movement and spatial redistribution. **A** Schematic representation of the coffee-ring effect and Marangoni effect and their depositional model. The dark blue arrows represent the direction of the flow field caused by evaporation under the coffee-ring effect and Marangoni effect. The red and yellow cells represent the donor and recipient that spatially redistribute under different conditions. **B** Experimental time-lapse droplet evaporation with a pinned air-water-solid interface on an agar surface. **C** The final experimentally observed spatial distribution of *E. coli* K12 cells (GFP) after evaporation with the coffee-ring effect. **D** The final experimentally observed spatial distribution of *E. coli* K12 cells (GFP) after evaporation with the Marangoni effect counteracting the coffee-ring effect.

We tested this hypothesis using droplet experiments that allowed us to control evaporation-induced passive cell movement. We used the bacterium *Escherichia coli* HB101 carrying the IncP α-type broad-host-range conjugative plasmid RP4 as the donor strain, where RP4 encodes for ampicillin (Amp), kanamycin, and tetracycline (Tet) resistance [8]. We used *E. coli* K12 carrying plasmid pNW33n as the recipient strain, where pNW33n is a non-conjugative plasmid that encodes for chloramphenicol (Chl) resistance and green fluorescent protein (GFP) [9]. Successful conjugation of RP4 thus results in recipient cells that express GFP and are resistant to all four antibiotics. We performed droplet experiments by mixing the donor and recipient at equal concentrations and depositing 2 µl liquid droplets of the mixture in different environments. We incubated the droplets under non-evaporative (NEV; placing the droplets into 1.5 ml microcentrifuge tubes with 100% RH), evaporative (EV; placing the droplets on agar plates and incubating them in a constant humidity chamber at 30% RH), and Marangoni convection (MC; placing the droplets on agar plates containing 1% polyethylene glycol (PEG) and incubating the plates in a constant humidity chamber at 30% RH) conditions. We expected that water flow would not be observed and cells would remain dispersed throughout the droplets under NEV conditions, while uneven evaporation would drive water and cells toward the perimeter (i.e., the’coffee-ring’ effect) due to capillary forces under EV conditions (Fig. 1A) [10]. Under MC conditions, we expected centripetal Marangoni flows to counteract the capillary-driven movement of water and cells toward the perimeter (Fig. 1A) [11, 12]. We tracked changes to the droplets using a Drop Shape Analyzer DSA25I (A. Krüss Optronic, Hamburg, Germany) and imaged the droplets using a Nikon A1RsiHD 25 confocal laser scanning microscope (Nikon, Tokyo, Japan) (see details in the Supplementary Information). We performed all of our experiments three times for each treatment (i.e., three droplets per treatment), with three analytical replicates per droplet. We further demonstrated that there is no loss in cell viability over the time-course of the experiments (Supplementary Table S1).

We found that incubation conditions (NEV, EV, and MC) indeed have a profound effect on the spatial distribution of cells within the droplets (Fig. 1 and Supplementary Fig. S1). For NEV conditions, cells remained dispersed throughout the droplets for the duration of the experiment. For EV conditions, the droplets rapidly evaporated (Fig. 1B) and cells were transported to the solid-liquid-air interface, resulting in concentrated cells at the perimeter that formed a biofilm-like mass (Fig. 1C and Supplementary Fig. S1). We did not detect active flagellar motility under EV conditions, indicating that cells were likely transported via evaporation-induced passive movement (Supplementary Video 1). For MC conditions, the addition of PEG altered the trajectories of cells, with a portion moving toward the solid-liquid-air interface at the perimeter and another returning to the center of the droplets as expected by centripetal Marangoni flows (Supplementary Video 2) [11, 12]. Overall, PEG successfully inhibited the ‘coffee-ring’ effect and led to more uniform spatial distributions of cells within the droplets (Fig. 1D and Supplementary Fig. S1).

**Fig. 2:**
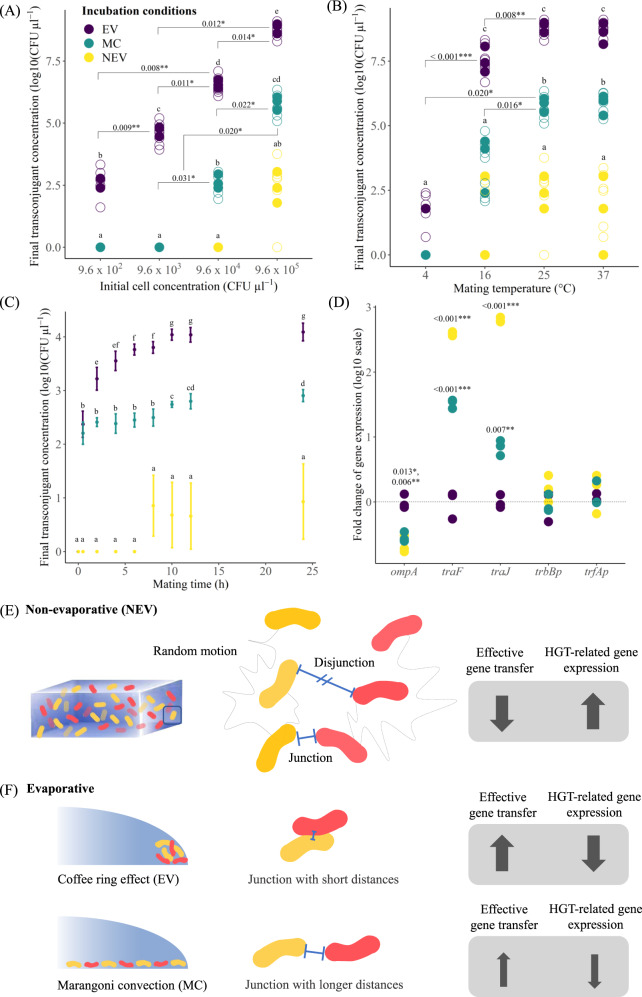
Experimentally measured effects of environmental conditions (evaporative, EV; Marangoni convection, MC; non-evaporative, NEV) on the extent of RP4 conjugation and the relative expression levels of conjugation-related genes. The effects of (**A**) the initial cell number, (**B**) mating temperature, and (**C**) mating time. For the initial cell number and mating temperature (**A** and **B**), experimental replicates (*n* = 3) are depicted as filled circles and analytical replicates (*n* = 3 per experimental replicate) are depicted as open circles. Some datapoints are not visible due to overlap with other datapoints. Statistically significant (≤0.05) Holm–Bonferroni adjusted *P* values are reported for relevant factor level comparisons (among levels of initial cell number or mating temperature within each evaporation condition). For all data (**A**, **B**, **C**), statistically significant differences between evaporation conditions are depicted with lower-case letters. **D** Relative expression levels of genes encoding conjugation-related membrane proteins (*ompA*), involved in mating pair formation (*trbBp* and *traF*) and involved with plasmid transfer and replication (*trfAp* and *traJ*). Expression levels are relative to those measured for EV conditions. Statistically significant (≤0.05) Holm–Bonferroni adjusted *P* values are reported for comparisons between the gene expression levels of the MC and NEV treatments against the EV treatment. The degree of statistical significance is indicated with *, ** or *** for *P* values that are 0.05–0.01, 0.01–0.001 or <0.001, respectively. Differences in the spatial distributions of cells and their cell-cell interactions under (**E**) non-evaporative and (**F**) evaporative conditions. Dotted lines in (**E**) represent bacterial trajectories of random motion and arrow thickness represents the magnitude of effective plasmid conjugation or conjugation-related gene expression.

We also found that incubation conditions (NEV, EV, and MC) had a profound effect on the extent of RP4 conjugation (Fig. 2 and Supplementary Tables S2 and S3). We first quantified the effects of initial cell number, mating time, and mating temperature on RP4 conjugation (Fig. 2A and Supplementary Fig. S2), finding that the extent of RP4 conjugation increased with increasing initial cell number (*F*_6,36_ = 71.90, *P* < 0.001; Supplementary Fig. S2A) and with mating temperature (*F*_6,36_ = 15.71, *P* < 0.001; Fig. 2B and Supplementary Fig. S2B). The extent of RP4 conjugation was significantly greater under EV than MC conditions already after 2 h mating time (Fig. 2C), while the extent of RP4 conjugation after 8 h mating time was 1000 times greater under MC than NEV conditions (Fig. 2C). Finally, the extent of RP4 conjugation after 10 h mating time remained approximately constant, being ~10^6^ times greater under EV than NEV conditions and 1500 times greater under MC than NEV conditions (Fig. 2A). These outcomes are consistent with our expectations, where the number and duration of cell-cell contacts are the highest under EV conditions leading to a higher extent of RP4 conjugation.

We finally quantified the expression of conjugation-related genes using qPCR (see details in the Supplementary Information). The membrane protein-encoding *ompA* gene, which can modulate conjugation rates [13], was expressed at significantly higher levels under EV and MC than NEV conditions (*F*_2,6_ = 31.72, *P* < 0.001; Fig. 2D and Supplementary Table S4). This agrees with a previous study demonstrating a positive relationship between *ompA* expression and conjugation rates [14]. However, genes involved with mating pair formation (*trbBp* and *traF*) [15, 16] and plasmid transfer and replication (*trfAp* and *traJ*) [17, 18] were not expressed or expressed at significantly lower levels under EV than NEV conditions (*traF* *F*_2,6_ = 1170.4, *P* < 0.001; *traJ* *F*_2,6_ = 1453.5, *P* < 0.001) (Fig. 2D and Supplementary Table S4). This contrasts with the common view that the extent of conjugative plasmid transfer is positively correlated with the expression of conjugation-related genes [14, 19] (although empirical evidence for such correlations is rare and may not exist for a multitude of dynamical reasons [20]). Mating pair formation and plasmid transfer and replication machinery are required to establish cell-cell junctions, replicate plasmids, and transfer plasmids to recipient cells [5, 8]. Under NEV conditions, cells can actively move and are unlikely to be collected at a fixed location (Supplementary Video 3). Such movements not only minimize the probability of forming cell-cell junctions but can also destabilize cell-cell junctions, thus requiring higher expression levels of these genes to achieve successful conjugation (Fig. 2E). In contrast, under EV conditions evaporation-induced passive cell movement results in larger numbers of cells pinned on surfaces or trapped at an air-water-solid interface. This results in a high-density biofilm-like mass with increased numbers and durations of cell-cell contacts. This, in turn, stabilizes cell-cell junctions and increases conjugation efficiency, thus requiring lower expression levels of these genes to achieve successful conjugation (Fig. 2F).

In conclusion, evaporation-induced passive cell movement can modulate the spatial distributions of cells with profound effects on the extent of plasmid conjugation. Our results demonstrate that evaporation-induced hydrodynamics can be an important determinant of plasmid transfer and should therefore be considered when predicting plasmid fate and designing strategies to control plasmid proliferation. Our results are potentially generalizable to a wide variety of conjugative plasmids and microbial communities, including those important for human health, the environment, and biotechnology.

## Supplementary information

Supplementary Video 1

Supplementary Video 2

Supplementary Video 3

Supplementary Information
