# Supplementary material for: Evaporation-induced hydrodynamics promote conjugation-mediated plasmid transfer in microbial populations
Source: ISME Commun. 2021 Oct 11;1:54. doi: 10.1038/s43705-021-00057-5 (PMC9723580; doi:10.1038/s43705-021-00057-5)
Supplement: Supplementary file 4 — Supplementary Information [file 43705_2021_57_MOESM4_ESM.pdf]

Supplementary Information

TITLE: Evaporation-induced hydrodynamics promote conjugation-mediated plasmid transfer in microbial populations

AUTHORS: Chujin Ruan[1, 2], Josep Ramoneda[2], Guowei Chen[3], David R. Johnson[2*], Gang Wang[1*]

AUTHOR AFFILIATIONS: [1]College of Land Science and Technology, China Agricultural University, Beijing, 100193, China; [2]Department of Environmental Microbiology, Swiss Federal Institute of Aquatic Science and Technology (Eawag), 8600 Dübendorf, Switzerland; [3]School of Civil and Hydraulic Engineering, Hefei University of Technology, Hefei, 230009, China

[*]**CORRESPONDENCE TO:**

Gang Wang, Email: Gangwang@cau.edu.cn

David R. Johnson, Email: david.johnson@eawag.ch

This file includes:

- Supplementary Materials and Methods
- Supplementary References
- Supplementary Figures S1-S2 and Tables S1-S4
- Captions for Supplementary Videos

Supplementary Materials and Methods

Bacterial strains and growth conditions. We used *Escherichia coli* HB101 carrying plasmid RP4 as the donor strain for the bacterial mating experiment, where RP4 is an IncP α-type conjugative plasmid carrying ampicillin (Amp), kanamycin (Km), and tetracycline (Tet) resistance genes. We verified the three antibiotic resistance abilities of *E. coli* HB101 by confirming growth with lysogeny broth (LB; 10 g/L tryptone, 5 g/L yeast extract, 10 g/L NaCl, pH 7.4) agar plates amended with 50 µg/mL Amp and 50 µg/mL Km. We routinely cultured *E. coli* HB101 under the same conditions to maintain RP4 stability. We used *E. coli* K12 carrying plasmid pNW33n (NCBI: txid226614) as the recipient strain, where pNW33n carries the chloramphenicol (Chl) resistance gene and thus differentiates it from *E. coli* strain HB101. We verified the chloramphenicol resistance ability of *E. coli* K12 by confirming growth on LB agar plates amended with 25 µg/mL Chl. We routinely cultured *E. coli* K12 under the same conditions to maintain pNW33n stability.

Enumeration of transconjugants from colony plating. For the first experiment, we set the initial cell concentrations of the donor (*E. coli* HB101):recipient (*E. coli* K12) (1:1) mix to 9.6×10^5, 9.6×10^4, 9.6×10^3 and 9.6×10^2 CFU/µL, deposited them onto LB agar plates, and kept the temperature constant at 25°C. After 8 h mating time, we cut the agar containing the cell deposits, transferred them to a 50-ml tube containing 20 mL of PBS solution, and vortexed the tube for approximately 5 min to resuspend the cells. We then quantified *E. coli* K12 transconjugants for each experimental replicate (n = 3) three times by selective plating of the suspended cells on LB agar plates supplemented with 50 µg/mL Amp, 50 µg/mL Km and 25 µg/mL Chl. For the second experiment, we fixed the initial donor (*E. coli* HB101):recipient (*E.

coli K12) (1:1) cell concentration to 10^5 CFU/µL and set the mating temperature to 4, 16, 25, or 37°C. We manipulated evaporation conditions and enumerated transconjugants after an 8 h mating time as described for the first experiment. For the third experiment, we set the donor (*E. coli* HB101):recipient (*E. coli* K12) mating time to 0.5, 2, 4, 6, 8, 10, 12, or 24 h with a fixed initial bacterial concentration of 10^5 CFU/µL and a constant mating temperature of 25°C. We again manipulated evaporation conditions and enumerated transconjugants as described for the first and second experiments. For all experiments, we replicated each treatment three times (i.e., three droplets) and quantified transconjugants three times for each droplet. Thus, we have a total of nine datapoints (three droplets x three analytical replicates). We averaged the analytical replicates prior to all statistical analyses to maintain independence. We verified that drying did not impact cell viability at the end of the experiment (Supplementary Table S1).

Microscopy. We centrifuged the overnight liquid cultures of donor (*E. coli* HB101) and recipient (*E. coli* K12) for 10 minutes at 6000 rpm with a high-speed centrifuge (H/T18MM, Herexi, China). We then discarded the supernatants and resuspended the cells in sterile phosphate-buffered saline (PBS). We next repeated the centrifugation and discarded the supernatants to eliminate any possible influence of extracellular secretions and residual culture medium. Finally, we resuspended the cells in sterile water with 1% polyethylene glycol (PEG) solution and adjusted the cell concentrations to obtain an optical density at 600 nm (OD_{600}) of 1 (≈10^8 CFU/mL) with a UV/VIS double beam spectrophotometer (A590, Aoyi, China). After the droplets evaporated, we imaged the droplets with a Nikon A1RsiHD 25 confocal laser scanning microscope (CLSM) (Nikon, Tokyo, Japan) with an emission wavelength of 488 nm. We used the 10× objective lens to image the entire droplet and the

100× objective lens to track the movement of individual cells under non-evaporative condition.

Quantification of the expression of outer membrane and conjugation transfer-related genes. For real-time quantitative PCR (qPCR), we cut the agar containing the cell deposits and transferred them to a 50-ml tube containing 20 ml of PBS solution. We then vortexed the tube for approximately 5 min and used tweezers to remove the remaining solid agar. We next collected the cells by low-speed centrifugation and resuspended them in 1 ml of PBS solution for RNA extraction. We extracted total RNA from the donor (*E. coli* HB101) and recipient (*E. coli* K12) mating samples using the *RTransZol* Up Plus RNA Kit (TransGen Biotech, Beijing, China). We reverse transcribed RNA into cDNA using *TransScript* II All-in-One First-Strand cDNA Synthesis SuperMix (TransGen Biotech, Beijing, China) for qPCR (One-Step gDNA Removal). The primer sequences for qPCR were according to Qiu *et al*[1] and Zhang *et al*[2]. The qPCR mixtures consisted of 10 µL 2×*TransStart* Top Green qPCR SuperMix (TransGen Biotech, Beijing, China), 0.4 µL forward primer (0.2 µM final concentration), 0.4 µL reverse primer (0.2 µM final concentration), 4 µL cDNA template, and 20 µL nuclease-free water. The thermocycling conditions for qPCR (Bio-Rad CFX96; Bio-Rad Laboratories, Hercules, CA) were 94°C for 30 s, followed by 45 cycles of 94°C for 5 s, annealing at 60°C for 15 s, 72°C for 10 s, and a final extension at 72°C for 1 min. We performed three analytical replicates for each experimental replicate.

Statistical analyses. We performed all statistical analyses between the means of analytical replicates for each experimental replicate to maintain independence We performed all statistical analyses using core functions in R[3] and generated plots using the package ggplot2[4].

For all datasets, we performed Analysis of Variance (ANOVA) with the number of transconjugants (CFU/µL) as a continuous response variable and evaporation conditions and initial cell density (first experiment), evaporation conditions and temperature (second experiment), and evaporation conditions and time point (third experiment) as categorical response variables. We performed repeated measures ANOVA for the latter to account for the repeated enumeration of transconjugants at different points in time. We tested for statistically significant differences between factor levels using the Welch two-sample two-sided t-test, which is suitable for comparisons between means of factor levels with unequal variances. We used a confidence threshold of 95% and adjusted the P-values for multiple comparisons using the Holm-Bonferroni method. We tested for the influence of evaporation conditions on the expression of HGT-related genes using one-way ANOVA and compared factor level means as described above. Finally, we measured the effect sizes of evaporative conditions (EV) over Marangoni convection (MC) on the total number of transconjugants detected after 8h mating time using the Cohen's d coefficient.[5]

Supplementary References

1. Qiu Z, et al. Nanoalumina promotes the horizontal transfer of multiresistance genes mediated by plasmids across genera. Proc Natl Acad Sci USA. 2012; 109: 4944-9.
2. Zhang Y, Gu AZ, He M, Li D, Chen J. Subinhibitory concentrations of disinfectants promote the horizontal transfer of multidrug resistance genes within and across Genera. Environ Sci Technol. 2016; 51: 570-80.
3. R Core Team. R: A language and environment for statistical computing. R Foundation for Statistical Computing, Vienna, Austria. URL https://www.R-project.org/. 2020.
4. Wickham H. Ggplot2: Elegant Graphics for Data Analysis. Springer-Verlag New York. ISBN 978-3-319-24277-4, https://ggplot2.tidyverse.org. 2016.
5. Lakens D. Calculating and reporting effect sizes to facilitate cumulative science: a practical primer for t-tests and ANOVAs. Frontiers in psychology, 4, 863. https://doi.org/10.3389/fpsyg.2013.00863. 2013.

Supplementary Figures

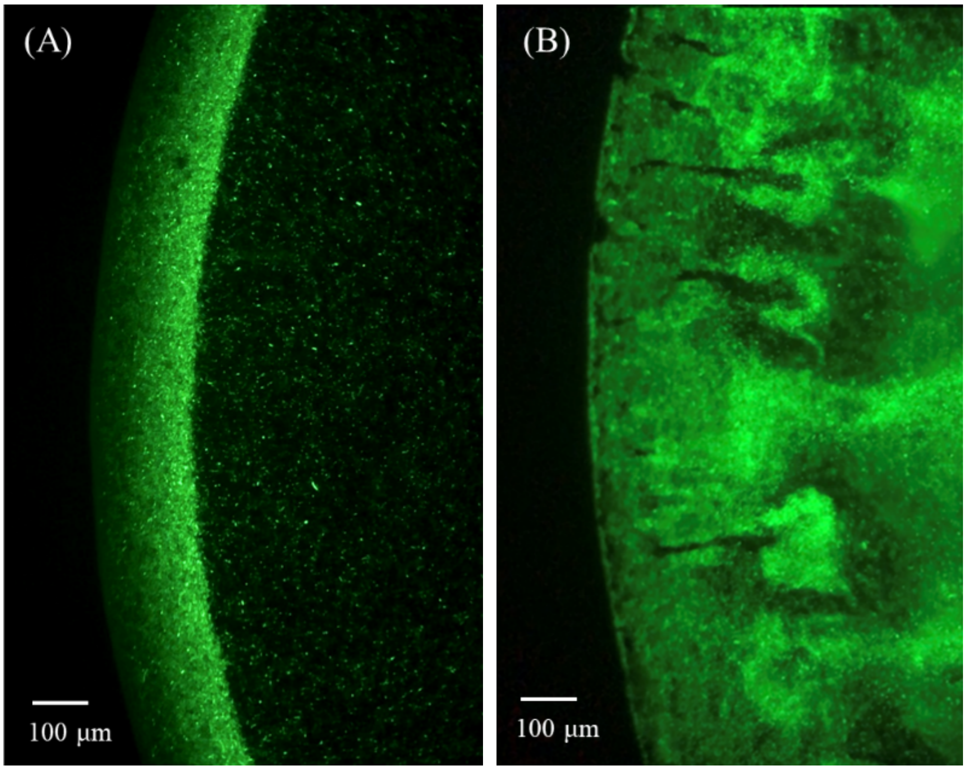

Supplementary Fig. S1: The perimeter of the droplet and the final deposition patterns of cells for different hydrodynamic conditions. (A) The final distribution of *E. coli* K12 cells (expressing green fluorescent protein) after evaporation with the coffee-ring effect. (B) The final distribution of *E. coli* K12 cells (expressing green fluorescent protein) after evaporation with the Marangoni effect. The initial cell concentration in the droplets was 10^5 CFU/μL.

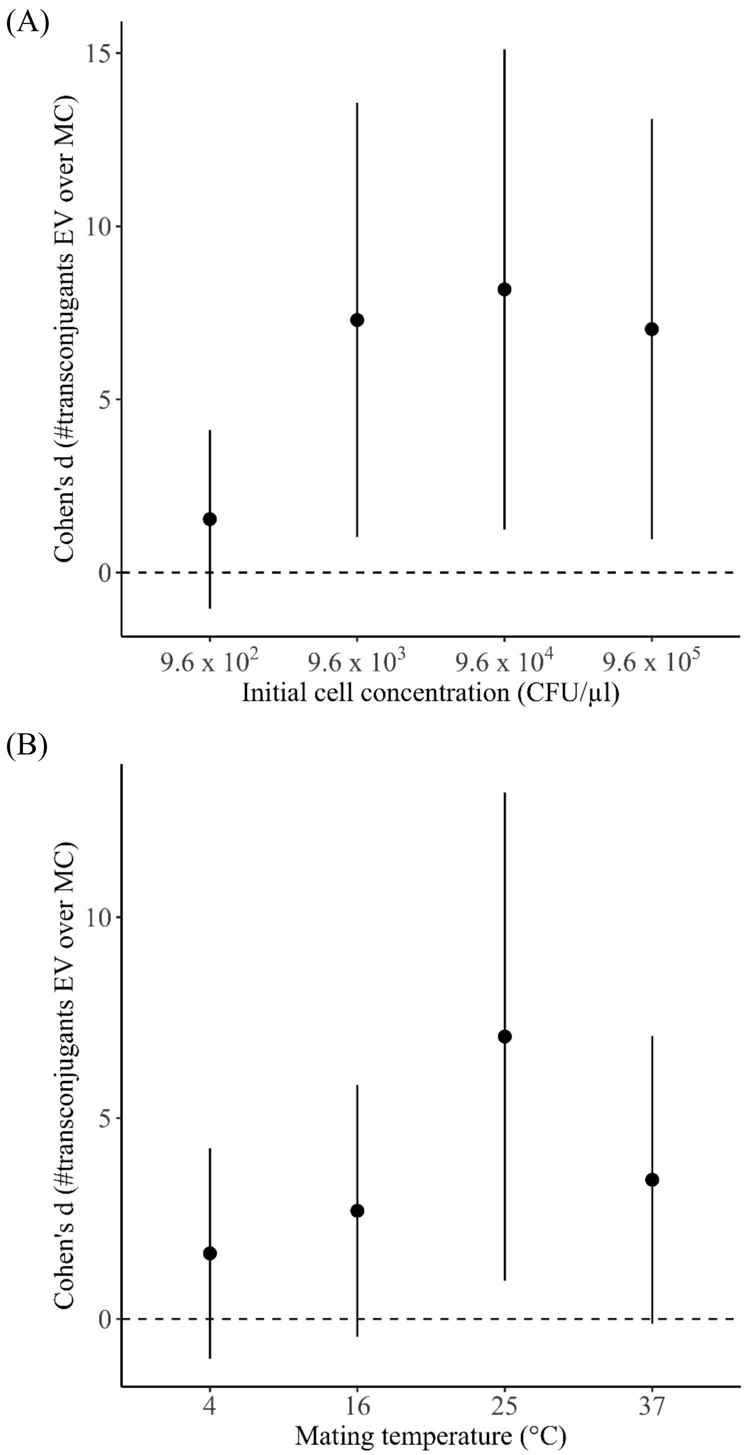

Supplementary Fig. S2: Effect sizes of evaporative conditions (EV) over Marangoni convection (MC) on the total number of transconjugants detected after 8h mating time. (A) Influence of the initial concentration of donor (*E. coli* HB101):recipient (*E. coli* K12) cells on effect sizes. (B) Influence of mating temperature on effect sizes. Effect sizes were estimated as Cohen's d coefficients, with 95% confidence intervals shown as error bars.

Supplementary Tables

Supplementary Table S1: Test of cell viability over the course of the plasmid conjugation experiment. The initial *E. coli* HB101 and K12 inocula were adjusted to an $OD_{600} \approx 1$ and mixed at equal volumes.

Experimental replicate	Analytical replicate	Cell concentration (× 10^8 CFU ml^{-1})	
		Start of the experiment (0h)	End of the experiment (24h)
1	1	2.2	3.6
	2	4.6	3.9
	3	4.2	2.5
2	1	3.9	2.8
	2	4.2	3.6
	3	2.7	2.6
3	1	1.5	3.5
	2	4.3	2.7
	3	5.2	3.9

Supplementary Table S2: ANOVA model summaries of the single and interactive effects of evaporation conditions with initial cell concentration, mating temperature, and time on the extent of plasmid RP4 conjugation. Df = degrees of freedom; statistical significance was set at P ≤ 0.05. Sums of squares were used to calculate the relative contribution of the different factors to the model and to calculate residual variation.

Experiment 1	*Df*	*Relative variance contribution (%)*	*F-value*	*P-value*
Evaporation	2	21.6	97.09	3.14×10^{-12}
Initial cell concentration	3	27.9	83.63	7.58×10^{-13}
Evaporation x Initial cell concentration	6	47.9	71.90	3.78×10^{-14}
Residual	-	2.7	-	-
Experiment 2				
Evaporation	2	48.4	81.75	1.93×10^{-11}
Mating temperature	3	16.5	18.60	1.88×10^{-6}
Evaporation x Mating temperature	6	27.9	15.71	2.94×10^{-7}
Residual	-	7.1	-	-
Experiment 3				
Evaporation (no time included)	2	13.8	39.01	1.81×10^{-12}
Residual (no time included)	-	86.2	-	-
Evaporation (time included)	2	50.0	444.35	$< 2.20 \times 10^{-16}$
Mating time	8	13.8	38.01	$< 2.20 \times 10^{-16}$
Evaporation x Mating time	16	23.8	33.16	$< 2.20 \times 10^{-16}$
Residual	-	12.5	-	-

Supplementary Table S3: Summary of the Welch two-sample two-sided t-tests comparing differences in the mean number of transconjugants between factor levels for experiments 1-3 (see Supplementary Table S2 above). The single and interactive effects of evaporation conditions with initial cell concentration, mating temperature, and time are shown. Confidence interval of 95%; Df = degrees of freedom; statistical significance set at $P \leq 0.05$; evaporative conditions = EV, Marangoni convection = MC, no evaporation = NEV. P-values were adjusted for multiple comparisons with the Holm-Bonferroni method.

Experiment 1	*Difference between means*	*P-value*
Evaporation		
EV - MC	1877.25	< 0.001
EV - NEV	1963.50	< 0.001
MC - NEV	86.25	< 0.001
Initial cell concentration		
9.6×10^5-9.6×10^4	2220.56	< 0.001
9.6×10^5-9.6×10^3	2425.89	< 0.001
9.6×10^5-9.6×10^2	2460.00	< 0.001
9.6×10^4-9.6×10^3	205.33	0.002
9.6×10^4-9.6×10^2	239.44	< 0.001
9.6×10^3-9.6×10^2	34.11	0.001
Experiment 2		
Evaporation		
EV - MC	3488.33	< 0.001
EV - NEV	3669.17	< 0.001
MC - NEV	180.83	< 0.001
Mating temperature		
37° - 25°	-463.33	0.954
37° - 16°	1302.78	0.008
37° - 4°	1998.33	< 0.001
25° - 16°	1766.11	< 0.001
25° - 4°	2461.67	< 0.001
16° - 4°	695.56	0.001
Experiment 3		
Evaporation		
EV - MC	5778.33	< 0.001
EV - NEV	6141.85	< 0.001
MC - NEV	363.52	< 0.001
Mating time		
24h - 12h	309.44	0.666
24h - 10h	622.78	0.625
24h - 8h	2069.44	< 0.001
24h - 6h	2358.89	< 0.001
24h - 4h	3148.89	< 0.001
24h - 2h	3821.11	< 0.001

24h - 0.5h	4366.11	< 0.001
12h - 10h	313.33	0.954
12h - 8h	1760.00	0.113
12h - 6h	2049.44	< 0.001
12h - 4h	2839.44	< 0.001
12h - 2h	3511.67	< 0.001
12h - 0.5h	4056.67	< 0.001
10h - 8h	1446.67	0.123
10h - 6h	1736.11	0.077
10h - 4h	2526.11	< 0.001
10h - 2h	3198.33	< 0.001
10h - 0.5h	3743.33	< 0.001
8h - 6h	289.44	0.768
8h - 4h	1079.44	0.132
8h - 2h	1751.67	0.006
8h - 0.5h	2296.67	< 0.001
6h - 4h	790.00	0.211
6h - 2h	1462.22	0.009
6h - 0.5h	2007.22	< 0.001
4h - 2h	672.22	0.070
4h - 0.5h	1217.22	< 0.001
2h - 0.5h	545.00	0.002

Supplementary Table S4: ANOVA model summaries of the effects of evaporation conditions on the expression levels of membrane and conjugation-related genes during mating. Df = degrees of freedom; statistical significance was set at $P \leq 0.05$.

Gene	*Df*	*F-value*	*P-value*
ompA	2	31.72	6×10^{-4}
trbBp	2	1.46	0.305
traF	2	1170.40	1.67×10^{-8}
trfAp	2	0.54	0.609
traJ	2	1453.50	8.74×10^{-9}

Captions for Supplementary Videos

Supplementary Video 1: The dynamic spatial redistribution of *E. coli* K12 cells in an evaporating droplet under the action of the coffee-ring effect. The initial cell concentration in the droplet was 10^5 CFU/µL. Video shown at 20× speed. The evaporation process was recorded with at an emission wavelength of 488 nm and 10× magnification.

Supplementary Video 2: The dynamic spatial redistribution of *E. coli* K12 cells in an evaporating droplet under the action of Marangoni convection. The initial cell concentration in the droplet was 10^5 CFU/µL. Video shown at 20× speed. The evaporation process was recorded at an emission wavelength of 488 nm and 10× magnification.

Supplementary Video 3: *E. coli* K12 cells' active movement and collision under non-evaporative conditions. The initial cell concentration in the liquid was 10^5 CFU/µL. Cell motility was recorded at an emission wavelength of 488 nm and 100× magnification.
